# Obesity Risk Factors Promote Metabolic Reprogramming and Viral Infection in Airways with Type 1 High Inflammation

**DOI:** 10.3390/biom15091229

**Published:** 2025-08-26

**Authors:** Paige Hartsoe, Niccolette Schaunaman, Taylor Nichols, Diana Cervantes, Stephanie Dawrs, Fernando Holguin, Hong Wei Chu

**Affiliations:** 1 Department of Medicine, National Jewish Health, Denver, CO 80206, USA; phartsoe11@gmail.com (P.H.); schaefern@njhealth.org (N.S.); taylordnichols@icloud.com (T.N.); dcervantes226@gmail.com (D.C.); 2 Department of Medicine, University of Colorado Anschutz Medical Campus, Aurora, CO 80045, USA; stephanie.dawrs@cuanschutz.edu (S.D.); fernando.holguin@cuanschutz.edu (F.H.)

**Keywords:** obesity, asthma, high-fat diet, palmitic acid, metabolic reprogramming, rhinovirus, interferon-gamma

## Abstract

Obesity is a significant health issue, as it is related to human diseases such as asthma and respiratory viral infections. Asthma patients with obesity have more severe diseases, which can be presented with type 1 (e.g., IFN-γ) high inflammation. The interactions of obesity or saturated fatty acids (e.g., palmitic acid, PA) with IFN-γ in airway viral infections have not been clear. In this study, we determined the role of obesity risk factors high-fat diet (HFD) and PA in rhinovirus infection in the context of IFN-γ stimulation in mice and cultured human tracheobronchial epithelial cells. We further examined the therapeutic effect of a glycolytic inhibitor on metabolic reprogramming and viral infection in our experimental models. In mice, HFD in combination with IFN-γ significantly increased lung rhinovirus levels as well as neutrophilic inflammation. Similarly, PA and IFN-γ combination increased viral infection in mice, but HFD or PA alone had a minimal effect on viral infection. Mouse model data were confirmed in cultured primary healthy human airway epithelial cells where PA and IFN-γ together increased viral load. Mechanistically, HFD or PA in combination with IFN-γ up-regulated the glycolytic pathway and generated metabolites favoring viral replication. Inhibition of glycolysis by 2-DG effectively reduced viral infection in human airway epithelial cells. Our data suggest that hosts with obesity along with type 1 high inflammation may be at an increased risk of respiratory viral infections. Intervention of the glycolytic pathway or its metabolites may reduce the severity of viral infection.

## 1. Introduction

Obesity (BMI ≥ 30) is a growing epidemic in the United States that puts a large burden on the healthcare system [1]. As of 2020, 42% of adults in the U.S. are obese [2]. Obesity is often associated with other health issues including diabetes, cardiovascular disease, non-alcoholic fatty liver disease, and cancer [3]. Obesity is also a major risk factor for asthma. In adults, the prevalence of asthma in obese individuals is 11% compared to 7% in their lean counterparts [4]. Asthma is a highly heterogenous disease which can be subcategorized into specific phenotypes or endotypes based on age of onset, allergic status, and clinical presentation. Obese asthmatic individuals often experience poor asthma control with conventional treatments such as glucocorticoids and are less likely to achieve remission with biological therapies [5]. Moreover, there are more frequent severe asthma exacerbations associated with respiratory viral infections in obese subjects with asthma. Viral asthma exacerbations induce high levels of interferon signaling including T helper 1 (Th1) or type 1 (T1) cytokine IFN-γ [6,7]. Importantly, increased T1 inflammation (e.g., more IFN-γ) has been observed in obese asthma patients [8,9,10]. Due to its ability to induce IFN-stimulated genes (ISGs), IFN-γ has been proposed to be beneficial in fighting viral infection [11]. However, excessive IFN-γ may promote viral infection and pro-inflammatory responses in airway epithelial cells in part through up-regulation of viral receptors such as ACE2 for SARS-CoV-2 [12] and intercellular adhesion molecule 1 (ICAM-1) for RV-A16 [13].

Metabolic dysfunction exists in obese subjects and can involve various metabolic pathways and processes. Elevated levels of free fatty acids (FFAs) in the plasma of obese individuals have been well documented and linked to insulin resistance and dysfunctional glucose metabolism [14,15]. Palmitic acid (PA) is the most common saturated fatty acid, accounting for 20–30% of fatty acids in the body [16]. The concentration of PA in the body is controlled through balancing dietary intake and endogenous production. An inactive lifestyle and increased carbohydrate consumption can elevate tissue PA levels. Accumulation of PA may lead to dyslipidemia and inflammation through Toll-like receptor 4 (TLR4) [16], G-protein coupled receptors (GPRs), and the NF-κB signaling pathway [17]. PA also has been shown to induce high levels of endoplasmic reticulum (ER) stress, increasing cellular dysfunction and death associated with exacerbation of pulmonary fibrosis [18]. There is strong evidence that PA disrupts cellular function and causes metabolic reprogramming through increased ceramide synthesis [19].

Viral infections are responsible for about 80% of asthma exacerbations [20]. Obese asthmatic individuals are at risk of more severe infections and poorer prognoses [21,22]. During the COVID-19 pandemic, obesity heightened the severity of illness and increased hospitalization rates [23]. Rhinovirus (RV) is a member of the *Picornaviridae* family responsible for the common cold. Major subgroups of the virus (e.g., RV-A16) infect cells through the receptor ICAM-1 [20]. RV-A1B is a minor group virus which infects airway epithelial cells through the low-density lipoprotein receptor (LDL-R) family of proteins [24]. RV infection may utilize glucose metabolism for enhanced viral replication [25].

How obesity risk factors (e.g., high levels of PA or high-fat diet, HFD) influence RV infection remains unclear. We hypothesized that high levels of PA or HFD in combination with excessive IFN-γ will worsen viral infection and inflammation in part through metabolic reprogramming. The aim of our study was to test this hypothesis by determining the effects of PA, HFD, IFN-γ, and rhinovirus on metabolites, viral infection, and inflammation in mouse models and human primary airway epithelial cell culture.

## 2. Materials and Methods

### 2.1. Virus Propagation

RV-A1B and RV-A16 (American Type Culture Collection, Manassas, VA, USA) were propagated in H1-Hela cells (CRL-1958, ATCC), purified, and titrated to obtain a plaque-forming unit (PFU), as described previously [26].

### 2.2. Mice

Wild-type (WT) C57BL/6 mice were purchased from the Jackson Laboratory (Bar Harbor, ME, USA) and housed in the Biological Resource Center at National Jewish Health (NJH) under pathogen-free housing conditions. All the experimental protocols were approved by the Institutional Animal Care and Use Committee at NJH (protocol #AS2792-03-26).

### 2.3. Mouse Model of High-Fat Diet (HFD) and IFN-γ Treatment

**Figure 1:**
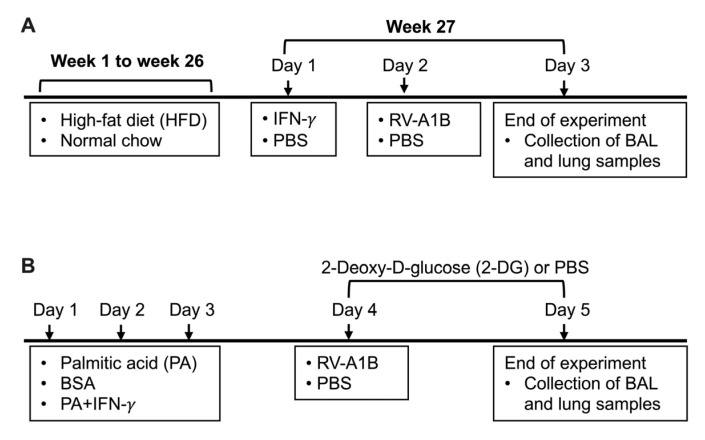
Timeline for mouse models receiving high-fat diet (HFD) and interferon-gamma (IFN-γ) treatment (**A**), and palmitic acid (PA), IFN-γ, and 2-Deoxy-D-glucose (2-DG) treatment (**B**) with or without rhinovirus-A1B (RV-A1B) infection. BSA, bovine serum albumin; PBS, phosphate-buffered saline.

To determine the effect of obesity and IFN-γ on viral infection and inflammation, we established a mouse model of obesity induced by HFD administration followed by IFN-γ treatment and RV-A1B infection (Figure 1A).

Starting at an age 9 weeks, mice (age and gender matched) were fed with either normal chow as a control (Inotiv, Indianapolis, IN, USA) or with HFD (Inotiv, Indianapolis, IN, USA) for 26 weeks. The HFD provided approximately 60% energy in the form of fat with an approximate fatty acid profile of 36% saturated, 41% monounsaturated, and 23% polyunsaturated fatty acid. Normal chow provided 22% energy in the form of fat containing 1.2% saturated, 1.7% monounsaturated, and 4.4% polyunsaturated fatty acid. Body weight was measured weekly. At week 27, mice were intranasally inoculated with 25 ng/mouse of recombinant mouse IFN-γ (PeproTech, Cranbury, NJ, USA) in 50 µL PBS [27] or PBS (control). Twenty-four hours post IFN-γ treatment, mice were intranasally inoculated with RV-A1B at 5 × 10^6^ PFU/mouse or PBS (control). Mice were euthanized by intraperitoneal injection of pentobarbital sodium (Fatal-Plus) in sodium chloride 24 h post infection. To obtain bronchoalveolar lavage fluid (BALF), 1 mL of sterile saline was used to lavage the lungs. BALF was used to prepare cytospin slides for leukocyte (e.g., neutrophils) differential counts using a Diff-Quick staining kit (IMEB INC., San Marcos, CA, USA). Cell differentials were determined as percentages of 500 counted leukocytes. The left lung was used for the measurement of viral load and host genes using qPCR. Cell-free BALF was used to measure proinflammatory mediators using ELISA, described below.

### 2.4. Mouse Model of PA, IFN-γ, and 2-DG Treatment

To examine the effect of PA on viral infection, we developed a model of infection under exogenous PA and IFN-γ conditions (Figure 1B). Mice (age and gender matched) were treated oropharyngeally with 100 µM PA (Sigma Aldrich, St. Louis, MO, USA) prepared in 0.1% fatty acid-free bovine serum albumin (BSA) or 0.1% fatty acid-free BSA-PBS (control) with or without intranasal inoculation with IFN-γ (PeproTech, Cranbury, NJ, USA) at 25 ng/mouse or PBS once daily for three consecutive days. This dose of PA was selected to represent a physiologically relevant concentration in obese individuals and based on our previous work [28,29,30]. On day 4, mice were intraperitoneally injected with 2-Deoxy-D-glucose (2-DG) (Sigma Aldrich, St. Louis, MO, USA) at 1000 mg/kg body weight [31] or PBS two hours prior to infection with RV-A1B. On day 5, mice received another intraperitoneal injection of 2-DG four hours prior to being euthanized, as described above.

### 2.5. Primary Human Airway Epithelial Cell Culture

Human tracheobronchial epithelial (HTBE) cells from healthy donors (n = 6) without history of lung disease or smoking were isolated as described in our previous publications [26,32]. Our study was approved by the Institutional Review Board (IRB) at NJH. HTBE cells were seeded into submerged culture on collagen-coated 24-well plates with a BronchiaLife medium (Lifeline Cell Technology, Frederick, MD, USA). At 80% confluency, cells were stimulated with 100 µM PA and 1 ng/mL recombinant human IFN-γ (PeproTech, Cranbury, NJ, USA) conjugated with 0.1% fatty acid-free BSA or 0.1% fatty acid-free BSA as a control. Twenty-four hours post treatment, cells were infected with RV-A16 at 5 × 10^4^ PFU/well. To determine a role of glycolysis in viral infection, 2-DG (Sigma Aldrich, St. Louis, MO, USA) at 5 mM was added to cells two hours prior to infection. After viral infection, fresh cell culture media with or without PA, IFN-γ, or 2-DG was added back. Forty-eight hours post infection, cells were lysed with RLT lysis buffer for reverse transcription and quantitative real-time PCR (RT-qPCR), and supernatants were collected for cytokine measurements. The dose of 2-DG was chosen based on the literature and our preliminary study where 2-DG at 5 mM was shown to reduce intracellular viral load by 70% with minimal cell death [33].

### 2.6. Measurement of L-Lactate

L-lactate levels in the cell culture supernatants were quantified using an L-lactate assay kit (RayBiotech, Peachtree Corners, GA, USA) according to the manufacturer’s instructions.

### 2.7. ELISA

CXCL10 (IP-10) was measured in the cell culture supernatants using a Human IP-10 DuoSet ELISA kit (PeproTech, Cranbury, NJ, USA) according to the manufacturer’s instructions.

### 2.8. Reverse Transcription and Quantitative Real-Time (RT-qPCR)

RV-A16 and ICAM-1 mRNA expression were measured by RT-qPCR on cell lysates from cultured airway epithelial cells. In the murine model, RV-A1B was measured from homogenized lung tissue using TRIzol to isolate RNA and from BALF using Mini Spin Columns (Epoch Life Science Inc., Missouri City, TX, USA) according to the manufacturer’s instructions. RNA was then reversely transcribed to cDNA using a Bio-Rad T100 thermocycler (Bio-Rad, Hercules, CA, USA).

Custom-made primers and probe (Integrated DNA Technologies, Coralville, IA, USA) for RV were 5′ CCTCCGGCCCCTGAAT 3′ (forward), 5′ GGTCCCATCCCGCAATT 3′ (reverse), and 5′ CTAACCTTAAAC CTGCAGCCA 3′ (probe). The mRNA levels of ICAM-1 were measured using a Taqman gene expression assay (Thermo Fisher Scientific, Waltham, MA, USA).

Gene expression was normalized to 18S RNA, and the comparative cycle of threshold (ΔΔCT) method was used to determine the relative level of gene expression.

### 2.9. Metabolomics

Cell supernatants and cell-free BALF of mice were collected to measure metabolites (e.g., glucose, lactate, D-glucose-6-phosphate, pyruvate, and D-ribulose-5-P) at the Mass Spectrometry Metabolomics Shared Resource Facility at University of Colorado Anschutz Medical Campus. To extract metabolites, cold 5:3:2 MeOH:ACN:H_2_O (*v*/*v*/*v*) solution was added in a 10:1 ratio to supernatants. Samples were vortexed vigorously for 30 min at 4 °C then centrifuged for 10 min at 18,213 rcf. Using 10 µL injection volumes, the supernatants were analyzed by ultra-high-pressure-liquid chromatography coupled to mass spectrometry (UHPLC-MS-Vanquish and Exploris, Thermo Fisher Scientific, Waltham, MA, USA). Metabolites were resolved across a 1.7 µm 2.1 mm × 150 mm Kinetex C18 column using a 5-min gradient previously described [34]. Following data acquisition, .raw files were converted to .mzXML using RawConverter. Metabolites were assigned based on intact mass, 13C isotope pattern, and retention times in conjunction with the KEGG database and an in-house standard library. Peaks were integrated using Maven (Princeton University). Quality control was assessed using technical replicates run at the beginning, end, and middle of each sequence, as previously described [35]. Raw peak data was normalized by median and used to generate figures.

### 2.10. Statistical Analysis

Statistical analysis was performed using GraphPad PRISM 10 software. For nonparametric data, a Mann–Whitney test for two group comparisons or Kruskal–Wallis test for multiple group comparisons was used. Parametric data for two-group and multiple-group comparisons were analyzed using a Student’s *t*-test or paired *t*-test and one-way ANOVA analysis, respectively. A *p* value < 0.05 was considered statistically significant.

## 3. Results

### 3.1. High-Fat Diet (HFD) in IFN-γ-Exposed Mice Increased Lung Viral Infection, Inflammation, and Glycolytic Metabolites

**Figure 2:**
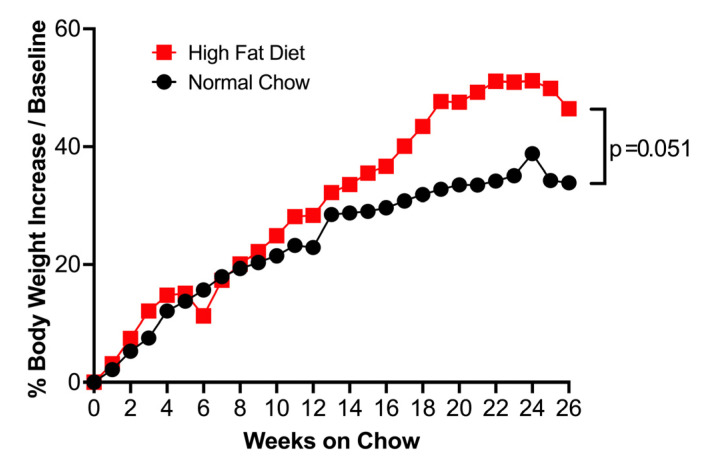
Mice fed with high-fat diet (HFD) for 26 weeks increased body weight gain, as indicated by the percentage of increase over starting weight at week 0 compared to mice fed with normal chow. Data represented as the median of n = 10 (normal chow) and n = 11 (HFD) mice. Red square = HFD, Black circle = normal chow.

After 26 weeks of HFD treatment, mice increased body weight as compared to those fed with normal chow (Figure 2).

**Figure 3:**
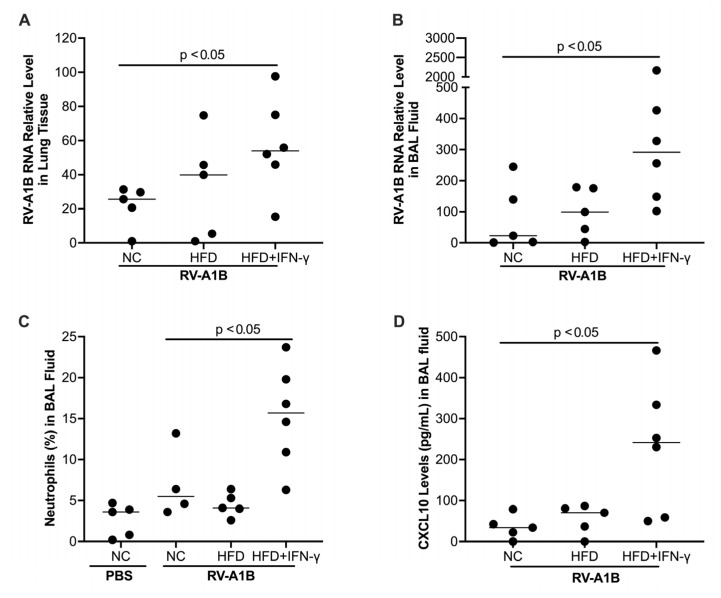
Mice fed with a high-fat diet (HFD) for 26 weeks and then exposed to interferon-gamma (IFN-γ) had increased viral levels (24 hr infection) in the lung tissue (**A**) and bronchoalveolar lavage (BAL) fluid (**B**), more neutrophil infiltration (**C**), and antiviral chemokine CXCL10 (**D**) compared to those fed with normal chow (NC) or HFD alone. Data is expressed as a median of n = 5–6 mice per group.

HFD treatment alone tended to increase viral levels in the lung tissue. Notably, mice fed with HFD and then treated with IFN-γ demonstrated significantly increased viral load in the lung tissue (Figure 3A) and bronchoalveolar lavage (BAL) fluid (Figure 3B) as compared to mice fed with normal chow. In line with the viral infection data, the levels of neutrophils were the highest in RV-infected mice with HFD and IFN-γ (Figure 3C). Additionally, RV-infected mice with HFD and IFN-γ treatment showed a significant increase in CXCL10, a chemokine involved in pro-inflammatory responses to viruses [33,34] (Figure 3D).

**Figure 4:**
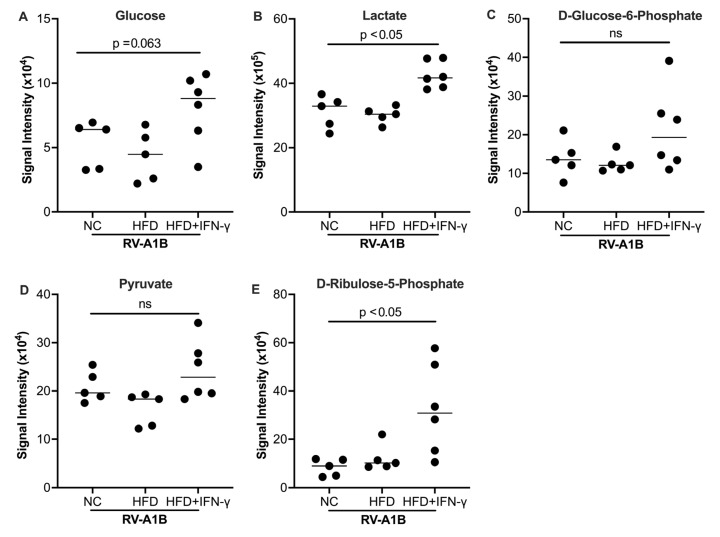
Effect of high-fat diet (HFD) and IFN-γ on glucose (**A**), lactate (**B**), D-glucose-6-phosphate (**C**), pyruvate (**D**), and D-ribulose-5-phopsphate (**E**) levels measured in the bronchoalveolar lavage fluid (BALF) of mice. Data is expressed as a median of n = 5 to 6 mice per group. ns, not significant.

Studies have shown that glycolysis and its derived metabolites promote viral infection or replication, including RNA viruses such as SARS-CoV-2 [35]. To determine the potential mechanisms of increased viral infection in RV-infected mice with HFD and IFN-γ, we measured metabolites known to contribute to viral infection. The combination of HFD and IFN-γ tended to increase D-glucose and significantly increased lactate (Figure 4A,B), two major glycolytic metabolites, in the BAL fluid of mice infected with RV. D-glucose-6-phosphate (downstream of D-glucose) and pyruvate trended higher (Figure 4C,D) in the combination group. Importantly, HFD, IFN-γ, and RV treatment significantly increased D-Ribulose 5-phosphate, a product derived from D-glucose-6-phosphate in the BAL fluid of mice (Figure 4E).

Together, our mouse model data demonstrated that HFD in combination with IFN-γ increased glycolysis and its pro-viral derivatives as well as susceptibility to RV infection coupled with lung neutrophilic inflammation.

### 3.2. Effects of Palmitic Acid (PA) on RV Infection, Inflammation, and Glycolysis in IFN-γ-Exposed Mice

**Figure 5:**
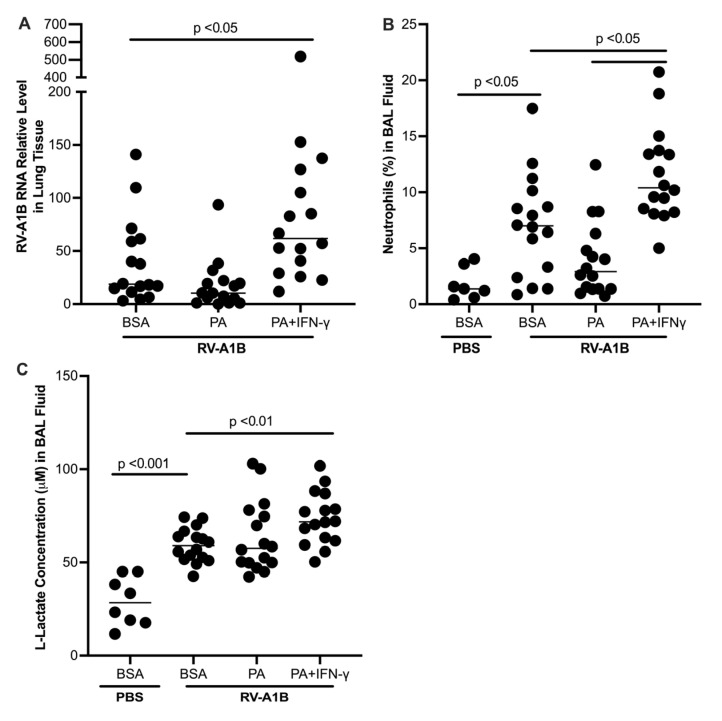
Prior exposure to palmitic acid (PA) and interferon-gamma (IFN-γ) increased viral levels in homogenized mouse lung tissue (**A**), as well as neutrophils (**B**) and L-lactate (**C**) in bronchoalveolar lavage (BAL) fluid. Data is expressed as a median of n = 7 to 16 mice per group.

As PA is the most abundant or common saturated fatty acid in the body and diet, we tested if PA may serve as a major contributor to viral infection. During RV infection, PA alone did not significantly alter viral load, but in combination with IFN-γ, it significantly increased viral load (Figure 5A). The increased viral load in PA and IFN-γ treated mice was coupled with increased neutrophil levels in BAL fluid (Figure 5B). Similar to the data observed in HFD-fed mice, L-lactate levels in BAL fluid were significantly upregulated by the combination of PA and IFN-γ in the presence of viral infection, suggesting increased glycolytic activity (Figure 5C).

### 3.3. Effects of a Glycolytic Pathway Inhibitor on Lung Viral Infection and Inflammation in PA-Treated and IFN-γExposed Mice

**Figure 6:**
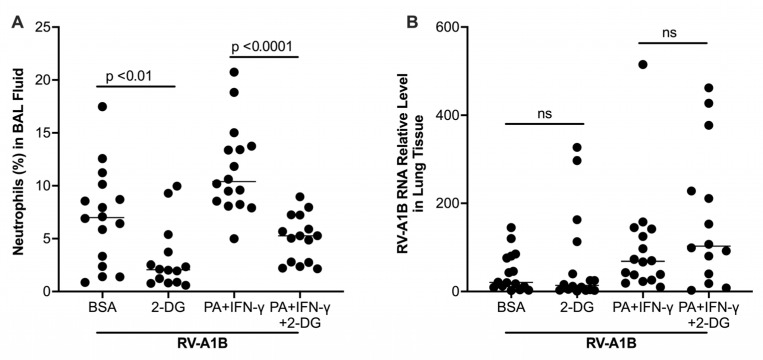
Effect of a glycolytic inhibitor 2-DG on airway inflammation and viral load in mice exposed to palmitic acid (PA) and interferon-gamma (IFN-γ). (**A**) Percentage of neutrophils in bronchoalveolar lavage (BAL) fluid of mice. (**B**) Viral load measured in homogenized lung tissue of infected mice. Data are expressed as a median of n = 13 to 16 mice per group. ns, not significant.

As glycolytic activity is elevated in PA-treated and IFN-γ-exposed mice with RV infection, we tested if 2-DG, an inhibitor of glucose conversion to glucose-6-phosphate via inhibition of hexokinase, attenuated viral infection. As shown in Figure 6A, 2-DG as compared with PBS control significantly reduced neutrophil influx into BAL fluid. However, 2-DG had no effect on viral load (Figure 6B).

### 3.4. PA in the Presence of IFN-γ Increased Viral Load in Cultured Human Primary Airway Epithelial Cells

**Figure 7:**
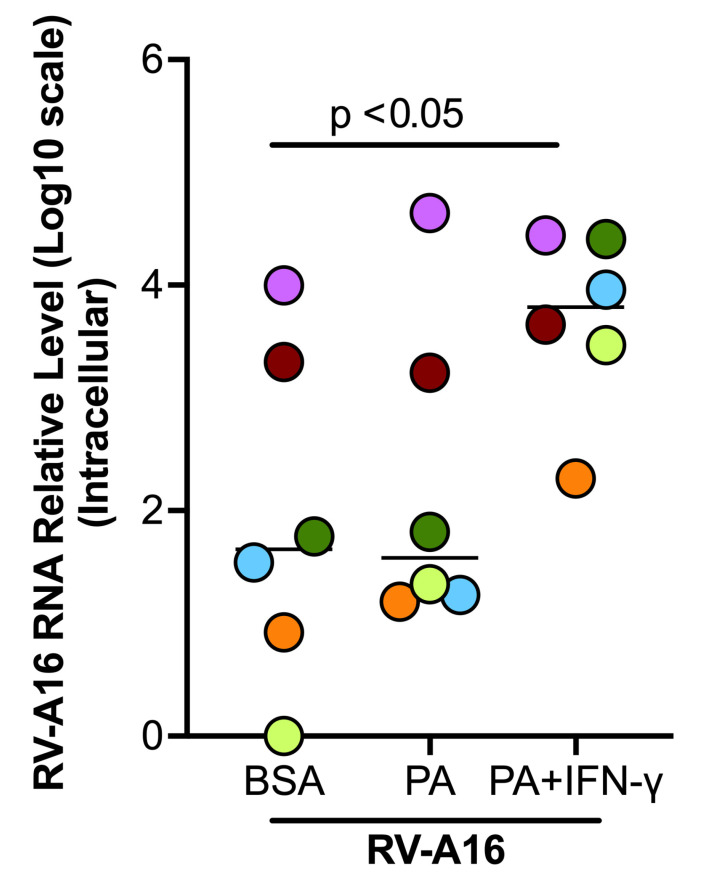
Palmitic acid (PA) and interferon-gamma (IFN-γ) increased viral load in cultured healthy donor tracheobronchial epithelial cells. Data are expressed as a median of n = 6 subjects. Each color indicates a human donor.

Having shown the effects of combined treatment of HFD or PA and IFN-γ on viral infection and inflammation in mice, we sought to test if PA and IFN-γ increase the severity of viral infection in primary human airway epithelial cells, the major type of cells infected by RV. Stimulation with PA alone did not alter viral load; however, co-stimulation of PA with IFN-γ significantly increased viral load compared to virus alone (Figure 7).

### 3.5. PA in the Presence of IFN-γ Increased Glucose Metabolism in Cultured Human Primary Airway Epithelial Cells

**Figure 8:**
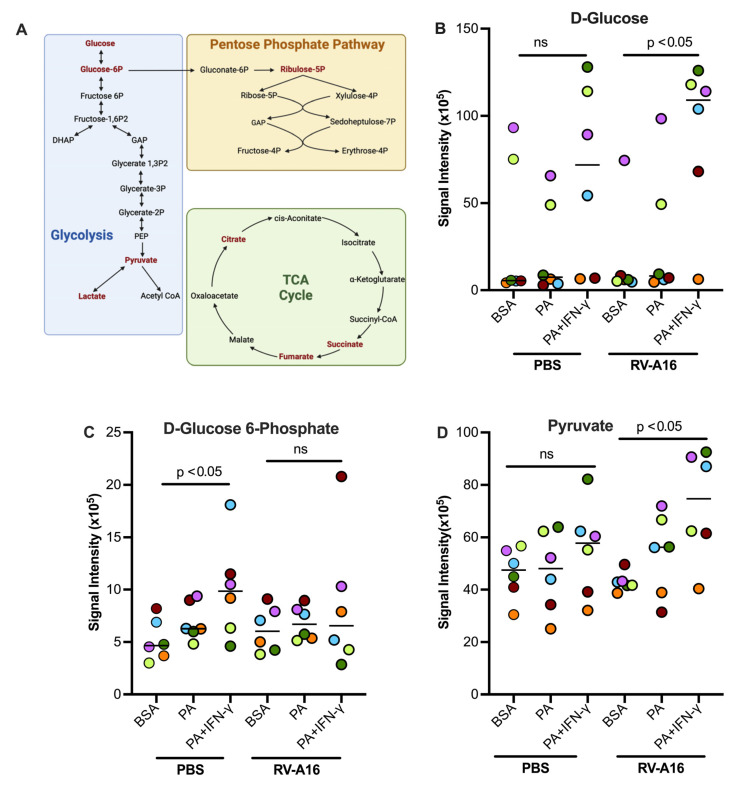
Palmitic acid (PA) and interferon-gamma (IFN-γ) in the presence or absence of rhinovirus RV-A16 alter cellular metabolism. (**A**) Diagram of glycolysis, the tricarboxylic citric acid (TCA) cycle, and the Pentose Phosphate Pathway. Dark red font = metabolites measured in our experiments. Created in BioRender. Schaunaman, N. (2025) https://BioRender.com/nn7xxtu. Glycolysis metabolites D-glucose (**B**), glucose-6-phosphate (**C**), and pyruvate (**D**) were measured in supernatants of cultured normal human tracheobronchial epithelial cells under submerged conditions. Data are expressed as a median of n = 6 subjects. Each color indicates a human donor. ns, not significant.

**Figure 9:**
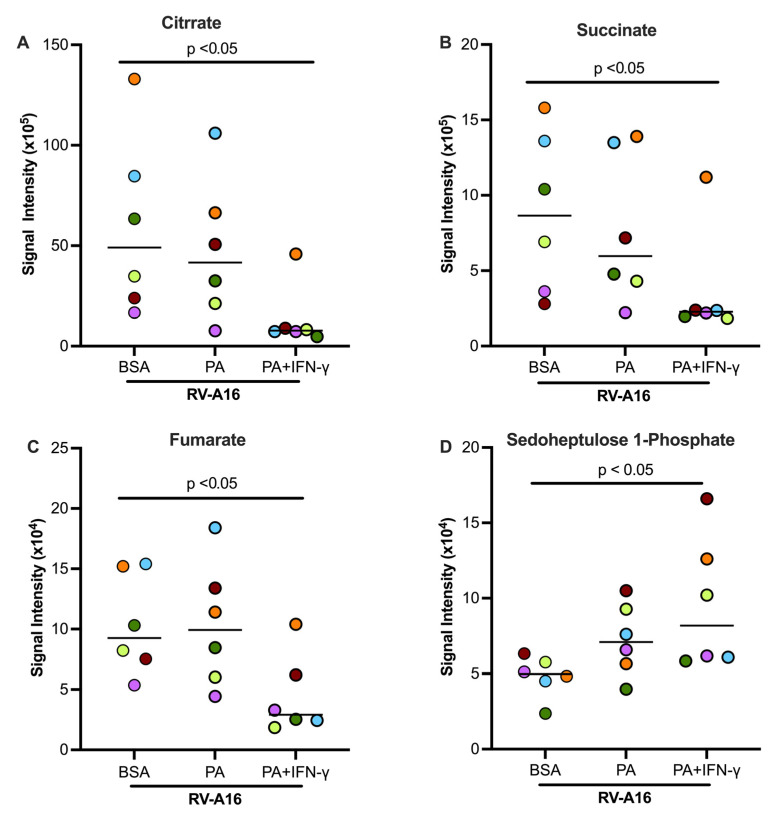
In supernatants of cultured normal human tracheobronchial epithelial cells, TCA cycle metabolites citrate (**A**), succinate (**B**), and fumarate (**C**) were downregulated by PA and IFN-γ during RV-A16 infection. Sedoheptulose-1-phosphate (**D**), a metabolite of the Pentose Phosphate Pathway, was upregulated. BSA, bovine serum albumin. Data are expressed as a median of n = 6 subjects. Each color indicates a human donor.

Figure 8A illustrates the glycolytic pathway, pentose phosphate pathway, and TCA cycle. Some key metabolites, as indicated by dark red, were measured in this study. In the absence of viral infection, PA and IFN-γ treatment tended to increase glucose and pyruvate but significantly increased the levels of D-Glucose 6-phosphate (Figure 8B–D) as compared to non-treated cells or those treated with PA alone. In viral infected cells, PA and IFN-γ did not further increase D-Glucose 6-phosphate despite D-glucose being significantly increased (Figure 8B). However, pyruvate, the end product of the glycolytic pathway, was significantly increased in RV-infected cells with co-treatment of PA and IFN-γ (Figure 8D). Unlike the glycolytic metabolites, levels of major TCA cycle metabolites including citrate, succinate, and fumarate were significantly decreased in infected cells treated with PA and IFN-γ (Figure 9A–C). Another metabolite, sedoheptulose-1-phosphate, a precursor to D-ribulose-5-Phosphate, was significantly upregulated (Figure 9D) by PA and IFN-γ in viral infected cells.

### 3.6. Blocking Glycolysis with 2DG Decreased Viral Load in Cultured Human Primary Airway Epithelial Cells

**Figure 10:**
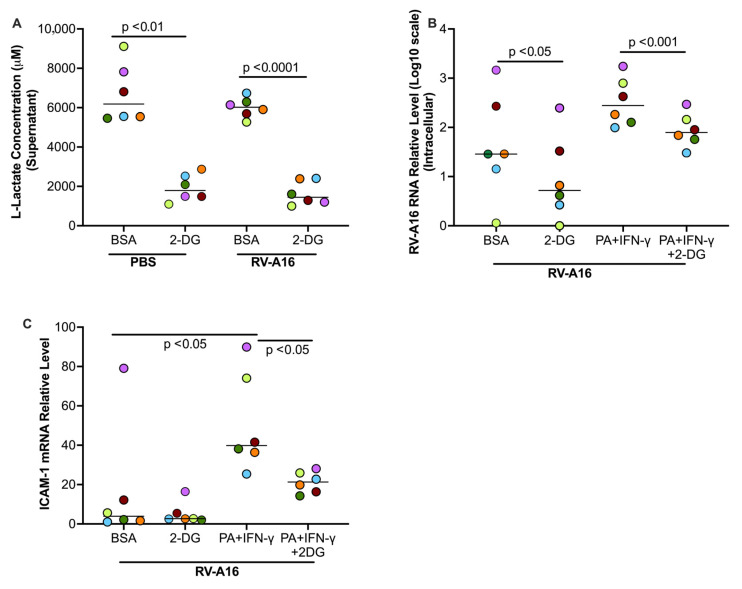
In cultured healthy donor tracheobronchial epithelial cells, 2-DG, an inhibitor of glycolysis, decreased the levels of L-lactate in supernatants (**A**) and RV-A16 (**B**) and RV-A16 receptor ICAM-1 (**C**) in the cells. BSA, bovine serum albumin. Data are expressed as a median of n = 6 subjects. Each color indicates a human donor.

We sought to determine if elevated glycolysis by PA and IFN-γ is responsible for increased viral infection by using 2-DG to inhibit the glucolytic pathway. To confirm if 2-DG was effectively inhibiting glycolysis, we measured the levels of L-lactate, an end product of glycolysis, in the supernatant. 2-DG treatment significantly decreased L-lactate production compared to the BSA control in non-infected and infected cells (Figure 10A). Notably, 2-DG was found to significantly decrease viral load in the absence and presence of PA and IFN-γ (Figure 10B). During viral infection, RV-A16 receptor ICAM-1 was up-regulated by PA and IFN-γ, which was also significantly down-regulated by 2-DG (Figure 10C).

## 4. Discussion

Viral infection in asthma including obese asthma continues to pose a significant health challenge, but the underlying mechanisms remain largely unknown. In the current study, we discovered that obesity risk factors HFD or PA in combination with IFN-γ significantly increased viral infection both in mouse models and in cultured human primary airway epithelial cells. Our research findings may have clinical implications to identify a subset of obese asthma subjects presenting a T1-high endotype at an increased risk of viral infection.

Metabolic abnormality or reprogramming is a salient feature of obesity [36,37]. Obesity is associated with excess in calories, usually obtained from a diet enriched with fat and sugar. Among the lipids, saturated fatty acids particularly PA have been shown to be increased in obesity [38]. The direct role of a HFD or PA in respiratory viral infection has not been well investigated. Here, we found that an HFD or PA alone trended to increase viral load in mouse lungs or human airway epithelial cells. A previous study [39] found that HFD-fed mice increased influenza A virus load and impaired the anti-viral inflammatory response in the lung. Why HFD or PA alone in our study did not significantly increase viral load remains unclear, but it may be related to the use of RV and the nature of our acute infection model. Notably, when HFD or PA combined with IFN-γ, a significant increase of viral infection occurred. This finding was surprising given the known role of IFN-γ in the antiviral response. IFN-γ signals through the JAK-STAT signaling pathway to induce interferon stimulated genes (ISGs) [40]. Multiple publications demonstrated the beneficial role of IFN-γ in host defense against other viruses including SARS-CoV-2, influenza A virus, and varicella-zoster virus [41,42,43,44], but the in vivo role of IFN-γ in RV infection has not been tested. However, excessive IFN-γ may promote viral infection and pro-inflammatory responses in epithelial cells in part through up-regulation of viral receptors such as ACE2 for SARS-CoV-2 [12] and ICAM-1 for RV16 [13]. Moreover, a mouse model study suggests that IFN-γ increases susceptibility to influenza A virus infection [45]. Thus, our study for the very first time demonstrated a detrimental role of interactions of saturated fatty acids and type 1 inflammation in lung defense against RV infection.

Obesity is a risk factor of SARS-CoV-2 infection and other viruses such as influenza [46,47,48]. However, the mechanisms by which obesity increases viral infection under different disease settings are poorly understood. SARS-CoV-2 have been shown to alter cellular metabolism and shift cells towards glycolysis to favor rapid energy production and viral replication [49]. Inhibition of glycolysis by 2-DG has been proposed as a potential antiviral therapeutic in rhinovirus and SARS-CoV-2 studies [33,50,51]. We found that glycolytic metabolite D-Glucose 6-phosphate was increased during viral infection in the presence of HFD or PA and IFN-γ, and inhibition of glycolysis by 2-DG significantly decreased viral load in human airway epithelial cells. Interestingly, D-Glucose 6-phosphate was coupled with increased ribulose-5P, suggesting that glycolytic products could be shunted into the pentose phosphate pathway (PPP) for nucleotide production to support viral replication. Future studies could further measure glycolytic activity using the Seahorse assay as well as explore the role of the PPP further. Benfooxythiamine could be used to inhibit transketolase, an enzyme that plays a role in the conversion of Glucose-6-phosphate to ribose-5-phosphate [52].

Besides the role of glycolysis/PPP in viral infection, other mechanisms may be involved. In this study, we examined the effect of PA and IFN-γ on the expression of ICAM-1, which has been shown as a major receptor for epithelial entry of RV-A16 [53,54]. Importantly, previous studies suggest that PA or IFN-γ may upregulate ICAM-1 expression [13,55], but the combinational effect of PA and IFN-γ has not been investigated. For example, IFN-γ was found to be a strong inducer of ICAM-1 expression in airway epithelial cells and could potentially enhance rhinovirus infection [13]. PA has been shown to trigger STING activation through the induction of mitochondrial damage and sequential mtDNA release, increasing ICAM-1 expression in cultured aortic endothelial cells [56]. Our data showing more ICAM-1 by PA and IFN-γ during viral infection are in line with previous studies and provide an additional mechanism of increased viral entry. Thus, to reduce viral infections in obese subjects with type 1 inflammation, potential therapeutics targeting viral entry via ICAM-1 and viral replication induced by increased glycolysis/PPP should be considered for future studies.

Multiple strengths in the current study were noted, including the discovery of increased viral infection by co-treatment of PA or HFD with IFN-γ and the contribution of glycolysis and PPP to viral infection and inflammation. Nonetheless, we realize several limitations to the current study. First, we did not perform a longitudinal clinical study to determine if obese asthmatics with a type 1 inflammation-high endotype have more frequent viral exacerbations than lean asthmatics. Second, we did not extensively study the role of other glycolytic metabolites such as lactate in viral infection. Third, after 2-DG treatment, we observed significant reduction of viral load in human airway epithelial cells or neutrophils in mice, but we did not see viral reduction in IFN-γ- and PA-exposed mice treated with 2-DG. While the exact mechanisms are not clear, we speculate that viral reduction may have already occurred before the time we collected the lung tissue, as viruses utilize glycolytic products early in the viral replication process [57]. As glycolysis has been shown to promote neutrophil migration and recruitment to the tissue [58], it is possible that inhibition of glycolysis reduced lung neutrophil levels after RV infection, which in turn impairs viral clearance since neutrophils may be necessary for an effective immune response against respiratory viral infection [59,60]. Thus, future clinical studies aimed at reducing glycolysis and viral infection need to be carefully designed to avoid the potential detrimental effect due to excessive inhibition of neutrophil recruitment into the lung. Fourth, the role of other metabolic pathways (e.g., lipids and amino acids) was not investigated in the current study. These additional studies are important to help us fully understand the role of metabolic reprogramming in viral infection in obesity and asthma and will be pursued in our future studies. Fifth, the mechanisms by which HFD and IFN-γ increased glycolysis such as increased D-glucose and lactate production in our RV-infected mice need to be further investigated. Nonetheless, previous studies suggest that HFD may increase the levels of lactate dehydrogenase (LDH) [61], an enzyme involved in lactate production. Interestingly, HFD could cause insulin resistance [62], which may increase D-glucose levels. IFN-γ has been shown to induce glycolysis and lactate production in part through hypoxia inducing factor-1alpha (HIF-1α) signaling [63]. Notably, RV infection has also been shown to increase IFN-γ production [64]. Together, it is possible that signaling pathways activated by HFD, IFN-γ, and RV infection interact to increase the activity of glycolysis. Lastly, we did not investigate whether glucose and other metabolites in cell culture supernatants or mouse bronchoalveolar lavage fluid may be transferred or transported from the cells, lung tissues, or the blood. This is indeed an important area for our future work to measure metabolites in the cells, lung tissues, or blood.

## 5. Conclusions

For the first time, we demonstrated that obesity risk factors such as excessive saturated fatty acids and high-fat diet increase human rhinovirus infection and neutrophilic inflammation under type 1 inflammation (e.g., more IFN-γ). Mechanistically, enhanced viral infection and inflammation may be in part attributed to metabolic reprogramming such as glycolysis induced by obesity risk factors and type 1 inflammation. Appropriate intervention of metabolic reprogramming may provide a new therapeutic approach to reduce the severity of viral infection in obese subjects with type 1 high inflammation asthma or other pulmonary diseases.

## Data Availability

The data presented in this study are available within the article.

## Abbreviations

BALF, bronchoalveolar lavage fluid; BMI, Body mass index; BSA, bovine serum albumin; HFD, high-fat diet; HTBE, human tracheobronchial epithelial; IRB, Institutional Review Board; LDL-R, lipoprotein receptor; PA, palmitic acid; PPP, pentose phosphate pathway; PFU, plaque-forming unit; RT-qPCR, reverse transcription and quantitative real-time PCR; RV, rhinovirus; Th1, T helper 1; T1, type 1, TLR4, toll-like receptor 4; 2-DG, 2-deoxy-d-glucose.
